# Enrichment and Purification of Aucubin from *Eucommia ulmoides* Ionic Liquid Extract Using Macroporous Resins

**DOI:** 10.3390/ma11091758

**Published:** 2018-09-18

**Authors:** Xinyu Yang, Mengxia Wei, Hao Tian, Tingting Liu, Lei Yang

**Affiliations:** 1 Key Laboratory of Forest Plant Ecology, Ministry of Education, Northeast Forestry University, Harbin 150040, China; iris_vincent@outlook.com (X.Y.); weimengxia@nefu.edu.cn (M.W.); 2 Institute of Agro-Poducts Processing Science and Technology, Yunnan Academy of Agricultural Sciences, Kunming 650221, China; tianhao.630@163.com; 3 College of Pharmacy, Qiqihar Medical University, Qiqihar 161006, China; ltting@outlook.com

**Keywords:** *Eucommia ulmoides*, samara, macroporous resin, aucubin, ionic liquid

## Abstract

Aiming to address the shortcomings of high-concentration ethanol or methanol extraction solutions that need to be diluted and concentrated prior to use in conventional macroporous resin adsorption approaches, an efficient approach for enrichment and purification of aucubin from the ionic liquid extraction solution of samaras of *Eucommia ulmoides* was proposed. Among the nine kinds of macroporous resins investigated, the HPD850 resin was found to be the most suitable. Equilibrium adsorption tests were investigated and found to be better fitted by the Langmuir isotherm model. After the dynamic tests on a column packed with HPD850, the optimum operational conditions were as follows: for the absorption process, an initial aucubin concentration of 9.87 mg/L, a sample volume of 13 bed volumes (BV), and a flow rate of 2 BV/h; for the water washing process, 5 BV of deionized water and a flow rate of 3 BV/h; for the ethanol desorption process, a 10–80% ethanol volume fraction as the eluent, 2 BV for each ethanol volume fraction, and a flow rate of 3 BV/h. The 40–80% ethanol volume fraction eluent was collected and concentrated to produce the final products, resulting in an aucubin purity and recovery of 79.41% and 72.92%, respectively.

## 1. Introduction

Liquid–liquid extraction using various solvents has been commonly used for the isolation of target analytes from solution, followed by a chromatographic process with a gradient or isocratic system [1]. Nevertheless, large solvent consumption, high energy consumption, high labor intensities, low recovery, and even safety problems, limit this method for large-scale industrial applications [2]. Therefore, a macroporous resin adsorption technology was developed for the enrichment and purification of target analytes from plant extracts. Macroporous resin used as an adsorbent can selectively adsorb components through hydrogen bonding and van der Waals interactions [3] based on the differences in physicochemical characteristics of the resins and target analytes. The adsorption and desorption process using macroporous resins exhibits improved characteristics such as high adsorption capacity, easy desorption and lower costs [4], which has led to its widespread application in the separation of targeted analytes from crude biological samples [5,6,7]. In previous studies, the adsorption processes by macroporous resins have been performed in aqueous solutions or dilute alcohol solutions of plant extracts to provide an efficient separation of components [7]. Better extraction yields can be achieved when using ionic liquids as the extraction solvents, but the separation of plant components from ionic liquid extraction solutions has not been reported in detail to the best of our knowledge.

**Figure 1:**
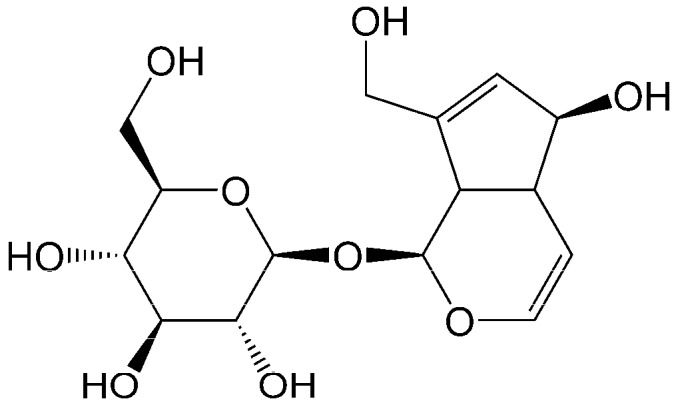
The molecular structure of aucubin.

*Eucommia ulmoides* are well-known deciduous trees with only a single species and genus belonging to the family of Eucommiaceae [8]. The samaras of *E. ulmoides* are an abundant source of pharmaceutically important iridoids (aucubin in particular). Indigenous to China, *E. ulmoides* is now widely distributed and is used for large-scale cultivation in China. The chemical structural of aucubin is shown in Figure 1. Aucubin, the predominant component in the samaras of *E. ulmoides* [9], has been reported to possess biological activities including anti-inflammatory activity [10], anti-fibrosis activity [11], suppression of oxidative stress [12], neuroprotection [9,13], hepatoprotection [14], promotion of angiogenesis [15], promotion of wound healing [16], and acceleration of the program of osteoblast differentiation [17]. Several techniques have been used to separate aucubin from *E. ulmoides* extracts, including aqueous two-phase extraction [18], column chromatographic procedures involving silica gel [19] or octadecylsilyl silica gel [20], and macroporous resin adsorption methods [20,21,22]. The macroporous resin adsorption approach has become mainstream for use in the separation of aucubin due to its simple operation, low-cost, and suitability for large-scale preparations. Due to the low aqueous solubility of aucubin, high concentrations of organic solvents such as ethanol or methanol was used in its extraction process [20,21,22], and supercritical CO_2_ was used as the extracting solvent and water-ethanol was used as the co-solvent in a few extraction processing [23]. Dilution and concentration of the extraction solutions are required before the macroporous resin adsorption treatment, which increases the labor intensity. Therefore, to maintain high extraction yields and to avoid the treatment of extraction solutions before absorption, ionic liquid is a desirable choice as the extraction solvent. Thus, finding an efficient method for the separation of aucubin from ionic liquid extraction solutions is of great importance.

The objective of this work was to develop an efficient approach for the enrichment and separation of aucubin from ionic liquid extracts of the samaras of *E. ulmoides*. To determine the most appropriate macroporous resin, nine different types of resins were investigated and compared according to their adsorption and desorption properties for the separation of aucubin. Dynamic adsorption and desorption experiments using the optimal resin were conducted to evaluate the technical parameters for the efficient separation of aucubin.

## 2. Materials and Methods

### 2.1. Materials and Chemicals

Samaras of *E. ulmoides* were obtained from Leshan (Sichuan, China) and authenticated by Prof. Yuangang Zu from the Northeast Forestry University in China. Dried samaras of *E. ulmoides* were pulverized using a grinder and were sieved (250–380 μm), then stored in closed vacuum desiccators at 4 °C in the dark prior to use.

Aucubin standard (purity ≥98%) was purchased from Shanghai Macklin Biochemical Co., Ltd. (Shanghai, China). Chromatographic grade acetonitrile was purchased from Thermo Fisher Scientific (Shanghai, China). 1-Butyl-3-methylimidazolium bromide (BmimBr) ionic liquid was purchased from Shanghai Chengjie Chemical Co. Ltd. (Shanghai, China) and used without further purification. The water used in all experiments was freshly prepared with a Milli-Q water cleansing apparatus (Millipore, MA, USA).

### 2.2. Pretreatment of Macroporous Resin 

Macroporous resins, including HPDD, HPD100A, HPD700, DM130, HPD750, HPD850, HPD400, HPD500 and HPD600 were obtained from Cangzhou Bon Adsorber Technology Co., Ltd. (Cangzhou, China). The physicochemical characteristics of these macroporous resins are listed in Table 1, including the surface area, average pore diameter, particle diameter, polarity and moisture content. The resins were stored in desiccators with deionized water to maintain constant moisture content. 

### 2.3. Preparation of Crude Ionic Liquid Extracts

Dried sample powder was mixed with 0.5 mol/L BmimBr solution with a liquid-solid ratio of 10 mL/g. The mixture was subjected to ultrasonic extraction for 30 min under 250 W of ultrasound power. This process was repeated twice, and the two resulting extraction solutions were combined to produce the raw extraction solution. The extraction solution was diluted using deionized water to form a series of aucubin concentrations (1.97–9.87 mg/L) for absorption, which were kept in the dark until use. 

### 2.4. HPLC Analysis and Quantification

Before HPLC determination, the aucubin standard solution and all the experimental solutions were filtered through 0.45 µm membranes. For qualitative and quantitative analysis, the HPLC determination of the contents of the crude BmimBr ionic liquid extract of the samples and standards was carried out using an Agilent 1260 Chromatographic system (Palo Alto, CA, USA), equipped with a quaternary pump, an on-line degasser, a diode-array detector, a thermostatic column compartment, and an autosampler. Ten microliters of samples and standards were run through an Agilent Zorbax Eclipse XDB-C18 reversed-phase column (Agilent, CA, USA; 4.6 mm × 250 mm, 5 μm) using 1 mL/min of acetonitrile: 0.1% formic acid in water 5:95 (*v*/*v*), with a thermostatic column of 25 °C and monitoring at 210 nm. Both retention times and spectral data were compared to their corresponding authentic standards. The concentration of aucubin in both samples and standards was calculated by the linear standard calibration curve: *Y* = 0.8693*X* − 0.1145 (*R*^2^ = 0.9993; *X* and *Y* represent the peak area and the concentration of aucubin, respectively) with a range from 0.002 mg/mL to 0.240 mg/mL. Each sample was done with three replicates to ensure accuracy.

### 2.5. Static Adsorption and Desorption Tests

#### 2.5.1. Adsorption Capacity and Desorption Ratio

The static adsorption experiments of the ionic liquid extraction solutions on various resins were performed as follows: 0.5 g of the resins (dry weight basis) and 30 mL of the ionic liquid extraction solution (known concentration) were added to a conical flask, which was capped and then shaken with 100 r/min at 25 °C for 24 h in a constant temperature oscillator (Changzhou Shangte Instrument Manufacturing Co. Ltd., Changzhou, China). The initial concentration of aucubin and its concentration after full absorption were detected by HPLC analysis. The adsorption capacity of resins was calculated as in Equation (1).

(1) $$Q_{e}=\frac{\left(C_{0}-C_{e}\right)\times V_{i}\left(1-M\right)}{W},$$

where *Q_e_* represents the adsorption capacity at equilibrium (μg/g anhydrous resin); *C*_0_ and *C_e_* represent the initial and equilibrium concentration of aucubin, respectively (mg/L); *V_i_* represents the sample solution volume before absorption (mL); *M* represents the moisture content of the resin (%); and *W* represents the mass of the resin (g).

For the static desorption process, removing the residual solution after adsorption equilibrium, the adsorbate-laden resins were first shaken (100 rpm) with 30 mL of deionized water for 24 h at 25 °C. Then, they were desorbed using 30 mL of a 90% ethanol aqueous solution for 2 h at 25 °C, with the flasks being shaken at <100 rpm. After the desorption process, the concentrations of aucubin in the desorption solutions were determined by HPLC. The desorption ratio of the resins was quantified according to Equation (2).

(2) $$D=\frac{C_{d}\times V_{d}}{\left(C_{0}-C_{e}\right)\times V_{i}}\times 100\%$$

where *C_d_* represents the concentration of aucubin in the desorption solution (mg/L); *V_d_* represents the desorption solution volume (mL); *D* represents the desorption ratio (%); and *C*_0_, *C_e_*, *V_i_* and *M* are the same as described above.

#### 2.5.2. Adsorption and Desorption Kinetics

The static adsorption and desorption kinetics of aucubin on the three preliminarily selected resins, HPD500, HPD600 and HPD850, were investigated. Based on the operational conditions in Section 2.5.1, the absorption procedure was continued for 8 h. In this process, 1 mL solutions were removed for HPLC analysis of the concentrations of aucubin after absorption for predetermined time intervals (0, 0.5, 1.0, 1.5, 2.0, 4.0 and 8.0 h). After reaching adsorption equilibrium, the desorption process was performed as described in Section 2.5.1. The concentration of aucubin in the desorption solutions after 0, 10, 20, 30, 40, 60, 90, 120 and 240 min, was monitored by removing 1 mL of the desorption solution for HPLC analysis.

#### 2.5.3. Effect of Extraction Solvent

Different concentrations of BmimBr ionic liquid (0.25, 0.50, 0.75 and 1.00 mol/L) and ethanol aqueous solution (20%, 40%, 60% and 80%) were used as the extraction solutions for the extraction of aucubin as described in Section 2.3. Then, the extraction solutions were used as the sample solutions for the absorption process to investigate the influence of the concentration of ionic liquid and ethanol solution on the absorption capacity of the resins. The residual solutions were filtered through 0.45 μm membranes and then analyzed by HPLC.

#### 2.5.4. Adsorption Isotherms

To evaluate the adsorption isotherms of aucubin on HPD850 resin, several experiments were conducted by introducing 30 mL sample solutions with different concentrations of aucubin (1.97, 3.95, 5.92, 7.90 and 9.87 mg/L) into conical flasks containing 0.5 g HPD850 resin (dry weight basis). The flasks were capped and then shaken (100 r/min) for 8 h at 5, 25 and 50 °C. The initial solutions and residual solutions after absorption were detected by HPLC to determine the concentration of aucubin. The adsorption isotherm of aucubin on the HPD850 resin was plotted by using the adsorption capacity versus the equilibrium concentration of aucubin. In addition, the Langmuir (Equation (3)) and Freundlich (Equations (4) and (5)) equations were used to fit the experimental values, and the degree of correlation between them was investigated.

Langmuir equation:

(3) $$Q_{e}=Q_{m}\frac{C_{e}}{\left(K+C_{e}\right)},$$

where *Q_e_* (mg/g) represents the concentration of aucubin per gram of anhydrous resin, namely, the adsorption capacity; *C_e_* (mg/L) represents the concentration of aucubin in solution at equilibrium; *K* represents the Langmuir constant; and *Q_m_* represents an empirical constant.

Freundlich equations:

(4) $$Q_{e}=KC_{e}^{1/n},$$

which was written in the linearized form as follows:

(5) $$lnQ_{e}=lnK+\frac{1}{n}lnC_{e},$$

where *K* represents the Freundlich constant, an indicator of adsorption capacity; and 1/*n* represents an empirical constant related to the magnitude of the adsorption driving force.

### 2.6. Dynamic Adsorption and Desorption Tests

A 12 mm × 500 mm open glass column (Sanaisi, Taixing, China) wet-packed with 10 g (dry weight basis) HPD850 was used for the dynamic adsorption and desorption tests, with a bed volume (BV) and a resin length of 15 mL and 18 cm, respectively. The sample solution with an appropriate concentration of aucubin was passed through the resin bed at different flow rates (2, 3 and 4 BV/h). The effluent was collected at 40 mL intervals, and the aucubin concentrations were determined by HPLC. Loading of the sample solutions was stopped upon reaching the breakthrough point. Because many impurities (ionic liquid and sugar) were present in the ionic liquid extraction solutions, the adsorbate-laden column was first washed with deionized water, and the concentration of ionic liquid in the water effluent was monitored by HPLC. The column was then eluted using different ethanol-water solutions (20%, 40%, 60% and 80%) at a flow rate of 2, 3 or 4 BV/h, respectively. The concentrations of aucubin in the effluent were detected by HPLC with the collection of solutions at 10 mL intervals.

### 2.7. Scale-Up Gradient Elution Tests

The scale-up gradient elution tests were conducted using a 70 mm × 1000 mm glass column wet-packed with 5 kg (dry weight basis) HPD850 resin, with a BV of 7.5 L. For the absorption test, a 97.5 L sample solution was passed through the resin bed at 2 BV/h. After that, the adsorbate-laden column was first washed with deionized water and then eluted with ethanol solutions of 10%, 20%, 30%, 40%, 50%, 60%, 70%, 80% and 90% in succession at a flow rate of 3 BV/h, using 2 BV for each volume fraction of ethanol. The effluent solution was collected at 5 L intervals, and the concentration of aucubin was determined by HPLC analysis. The recovery yield of aucubin was calculated using Equation (6):

(6) $$Y=\left[\frac{C_{d}\times V_{d}}{\left(C_{0}-C_{a}\right)\times V_{p}}\right]\times 100,$$

where *Y* (%) represents the recovery yield of aucubin; *C_a_* represents the concentration of aucubin in the eluent after absorption (mg/L); *V_p_* represents the processing volume of the sample solution (mL); and *C*_0_, *C_d_* and *V_d_* are the same as described above.

## 3. Results

### 3.1. Static Adsorption and Desorption Tests

The adsorption capacity and desorption ratio of macroporous resins are commonly regarded as two crucial factors for selecting a suitable resin, which depend on the physical and chemical properties (pore structure and diameter, surface functional group, etc.). Therefore, in this work, nine types of macroporous resins (HPDD, HPD100A, HPD700, DM130, HPD750, HPD850, HPD400, HPD500 and HPD600) were chosen for comparison to select the most appropriate resin in terms of the adsorption capacity and desorption ratio of aucubin.

#### 3.1.1. Adsorption Capacity and Desorption Ratio

The absorption capacities of aucubin on different macroporous resins are presented in Table 1. The results indicate that the adsorption capacities of aucubin on HPDD, HPD850, HPD500, and HPD600 were considerably higher than that of the other resins, with adsorption capacities higher than 140 μg/g. In general, resins with similar polarity to target analytes, larger surface area and larger average pore diameter are conducive to the absorption process [24], which may be the reason for the higher adsorption capacity of aucubin on these resins. Furthermore, the desorption ratios of aucubin on these macroporous resins were also investigated, and the results are shown in Table 1. As noted, HPD500 and HPD850 resins had a clear advantage over the others for the desorption of aucubin due to their higher desorption ratios (above 75%). The desorption ratio is highly related to the affinity between the analytes and resins, which depends on the physical force, (for example, van der Waals force) [25]. Thus, the HPD450, HPD850 and HPD500 resins might have a lower power for holding aucubin. In addition, desorption ratios are also influenced by the average pore diameter of resins, specifically, large average pore diameters will lead to higher desorption ratios. We hypothesized that these factors might explain the high desorption ratios of HPD850 and HPD500.

Considering both their absorption capacity and desorption ratio, the HPD500, HPD600 and HPD850 resins performed better for the separation of aucubin; thus, these three resins were chosen for further investigation in the following experiments.

#### 3.1.2. Adsorption and Desorption Kinetics Curves

Comparison of the adsorption capacity and desorption ratio of the macroporous resins is not sufficient for choosing the most suitable resin. Therefore, further experiments were conducted to investigate their adsorption and desorption rates.

**Figure 2:**
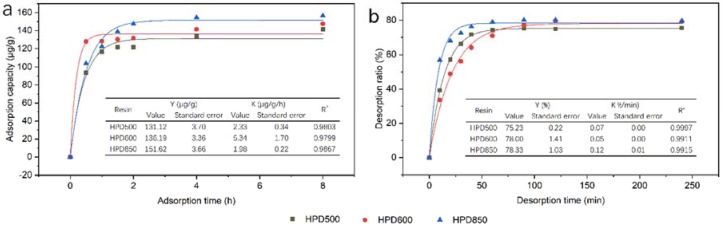
The adsorption kinetics curves (**a**) and desorption kinetics curves (**b**) for aucubin on HPD500, HPD600 and HPD850.

The adsorption kinetics for aucubin on HPD500, HPD600 and HPD850 resins are shown in Figure 2a; the adsorption capacity of aucubin increased markedly with the increase in adsorption time and reached equilibrium at approximately 4 h on each of the three resins. For all three resins, an initial rapid enhancement of the adsorption capacity was followed by a slow improvement, with adsorption times increasing from 0 to 0.5 h and 0.5 to 4 h, respectively. Although HPD600 had a higher adsorption capacity of aucubin than HPD850 at 0.5 h, the adsorption capacity of aucubin on HPD850 at equilibrium was significantly higher than on other resins. Thus, HPD850 demonstrated a better adsorption rate for aucubin than HPD500 and HPD600. In Figure 2b, the desorption kinetics for aucubin on HPD500, HPD600 and HPD850 resins are plotted. The results show that the desorption ratio of aucubin on HPD850 and HPD500 resins gradually increased as the adsorption time increased to 60 min (equilibrium). In contrast, the adsorption time for desorption ratio to reach equilibrium on HPD600 was extended to 90 min. Compared with HPD500, a higher desorption ratio of aucubin was achieved by HPD850 resin at equilibrium. Thus, HPD850 resin had a more satisfactory desorption rate for aucubin than the others.

In general, HPD850 resin had the best performance among these resins for the separation of aucubin, based on the adsorption and desorption kinetics curves. Thus, HPD850 was chosen as the appropriate resin for the following experiments. For static adsorption and desorption, 4 h and 60 min were selected as the adsorption and desorption times for aucubin on the HPD850 resin.

### 3.2. Influence of the Extraction Solvent

**Figure 3:**
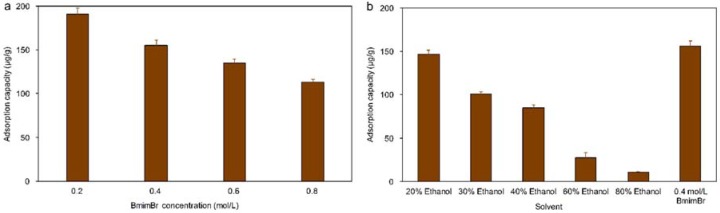
The effect of concentration of BmimBr ionic liquid (**a**) and ethanol (**b**) on the absorption capacity of aucubin on HPD 850.

Macroporous resins generally performed better for absorption of the target components in water or diluted ethanol solution, but the low solubility of aucubin in water or diluted ethanol solutions leading to its extraction was accomplished by a high volume fraction of ethanol, thus the direct absorption of aucubin from high-concentration ethanol extraction solutions to be inefficient. Thus, the effect of various concentrations of ethanol and BmimBr solution on the adsorption capacity of aucubin on HPD850 resin was investigated, the results of which are presented in Figure 3.

As shown in Figure 3, the adsorption capacity of aucubin decreased gradually as the concentration of BmimBr or ethanol decreased. However, a larger influence on the adsorption capacity was observed when the ethanol solution was diluted than when the BmimBr concentration was reduced. When using an 80% ethanol solution as the solvent for the extraction of aucubin, an additional dilution was required before absorption, which increased operation complexity and caused lower solubility of the target components. As shown in Figure 3b, 0.5 mol/L BmimBr gave a higher adsorption capacity than all concentrations of ethanol solution that were used. Here, the BmimBr extraction solution could be used for direct absorption without additional dilution. The adsorption capacity of the 0.25 mol/L BmimBr, solution was higher than that of the 0.5 mol/L BmimBr, solution, but the extraction result of the 0.5 mol/L BmimBr solution was better than that of the 0.25 mol/L solution because the high concentration of ionic liquid is more conducive to the extraction of target components [26]. Therefore, BmimBr at 0.5 mol/L provided the most efficient extraction and absorption results.

### 3.3. Adsorption Isotherms

**Figure 4:**
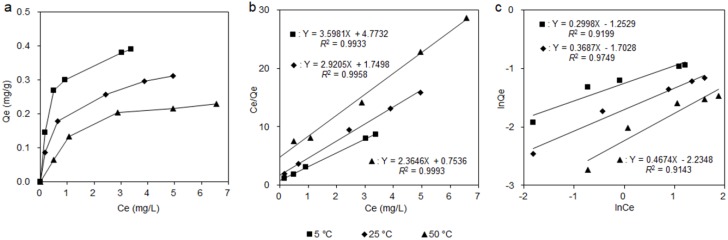
The adsorption isotherms of aucubin (**a**), the fitting result by Langmuir equation (**b**) and Frendlich equation (**c**) on HPD750 at different temperatures.

The adsorption behavior of the HPD850 resin for aucubin from ionic liquid extraction solutions with different initial concentrations (1.97, 3.95, 5.92, 7.90 and 9.87 mg/L) was investigated at different temperatures (5, 25 and 50 °C), and the corresponding results are shown in Figure 4. With increasing initial aucubin concentrations, the adsorption capacity was enhanced correspondingly for the three temperatures tested. The initial aucubin concentration of 9.87 mg/L in the sample solution was used for subsequent experiments. For the variation in temperature, lower temperatures were clearly more conducive to absorption. The adsorption capacity decreased with the increase in the temperature, which implied that the adsorption process was exothermic. The cooling process required a great deal of energy in actual practice, thus a median 25 °C was found to be more suitable and was selected for use in further experiments.

The Langmuir and Freundlich isotherm models (the two best-known models for describing the adsorption process) were used to evaluate the adsorption mechanism for aucubin on the HPD850 resin. The fitted results and parameters for the Langmuir and Freundlich models are shown in Figure 4 and in Table 2. The result of fitting the Freundlich model revealed that a 1/*n* value between 0.1 and 0.5 implied effective adsorption, a 1/*n* value between 0.5 and 1.0 indicated almost no absorption, and a 1/*n* value higher than 1.0 indicated that no absorption occurred [27]. As shown in Table 2, the 1/*n* values for aucubin using the Freundlich equation were between 0.1 and 0.5, indicating an effective adsorption of aucubin on HPD850. However, the correlation coefficients *R*^2^ values for the Freundlich equation were lower than that of the Langmuir equation (above 0.99), which illustrated a better fitting by the Langmuir equation. Therefore, the Langmuir equation can best be used to accurately describe and interpret the adsorption behavior of aucubin on HPD850.

### 3.4. Dynamic Adsorption and Desorption Test

#### 3.4.1. Dynamic Leakage Curves on the HPD850 Resin

**Figure 5:**
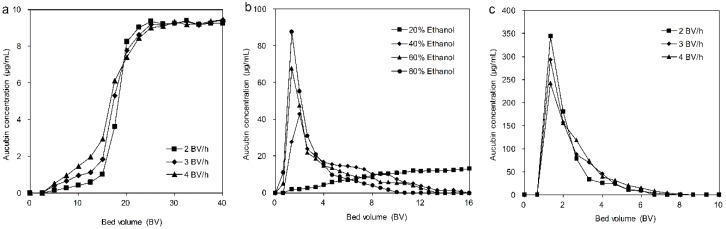
Dynamic process on columns packed with HPD850. Breakthrough curves of aucubin with different flow rates (**a**); desorption curves of aucubin with different ethanol concentrations (**b**); desorption curves of aucubin with different flow rates (**c**).

For the dynamic absorption process, the break-through point of the sample solution is commonly used to indicate the absorption saturation [3,27] because the target analytes rapidly leak from the resin after the break-through point. Thus, the dynamic leakage curve of aucubin on the HPD850 resin was investigated at different flow rates (2, 3 and 4 BV/h), with concentrations of aucubin in eluent solutions versus the loading volume of the sample solutions (Figure 5a). At a flow rate of 2 BV/h, the processing volume of the sample solution was larger than those at the 3 and 4 BV/h rates, from which we speculated that sufficient particle diffusion was obtained at the lowest flow rate. To simultaneously reach better absorption and reduce the waste of the sample solution, 2 BV/h was selected as the flow rate for use in the following tests, and the loading volume of the sample solution was 195 mL (13 BV).

#### 3.4.2. Dynamic Water Washing Process for the Ionic Liquid

Before desorption using ethanol solution, the adsorbate-laden column was washed with deionized water to remove impurities (such as sugar, salts and protein) and the ionic liquid, thus providing a foundation for the further recycling and reuse of the ionic liquid. Through detecting the water elution by HPLC, ionic liquid could be efficiently washed by deionized water, which illustrated that the ionic liquid was absorbed on the surface of the resins and that no intermolecular forces between the ionic liquid and resin were formed. When using 5 BV of deionized water for washing the resins, almost 86.93% of the HPD850 resin was recovered. Therefore, 5 BV of deionized water was used to wash the adsorbate-laden column before ethanol desorption in the following experiments.

#### 3.4.3. Dynamic Desorption on the HPD850 Resin

When adsorption equilibrium was achieved, the adsorbate-laden column was first washed with 5 BV of deionized water and then eluted using an ethanol solution with various concentrations (20%, 40%, 60% and 80%) and flow rates (2, 3 and 4 BV/h). The desorption curves were plotted against the concentration of aucubin in the eluent solution versus the volume of the desorption solution.

The desorption curves using different ethanol volume fractions are presented in Figure 5b, from which we can see that the desorption ratio of aucubin increased with the ethanol volume fraction increasing from 20% to 80%. For the ethanol volume fractions (40%, 60% and 80%), peaks containing high aucubin concentrations were produced, and the peak of the 80% ethanol volume fraction was higher than that of the other ethanol volume fractions. In contrast, no peak was formed for the 20% ethanol volume fraction, which revealed that the desorption ability of the 20% ethanol solution was not enough to efficiently desorb the aucubin from the resins. When using an ethanol volume fraction of 80%, aucubin was completely desorbed with a solvent volume of 150 mL. In comparison, at the ethanol volume fractions of 40% and 60%, the desorption of aucubin was basically finished, with a solvent volume of 230 and 190 mL, respectively. Although the ethanol volume fraction of 80% performed most efficiently in the desorption of aucubin from the HPD850 resin, it was also accompanied by the elution of a number of impurities, which influenced the purity of the recovered aucubin. Therefore, ethanol volume fractions of 60% were used as the desorption solutions in subsequent tests.

As shown in Figure 5c, the desorption curves using different flow rates indicated that aucubin required 100 mL of desorption solution to be eluted from the resins at 2 BV/h, but the desorption solution was increased to 110 mL for both 3 and 4 BV/h. Furthermore, the desorption result of 2 BV/h was better than the other two flow rates, as evidenced by the peak height. The lower flow rate was conducive to adequate contact between the desorption solution and the target analytes. Even so, the longer time consumption for the desorption process led to its lower efficiency. To simultaneously improve desorption efficiency and ensure the best desorption result, a flow rate of 3 BV/h was selected for further experiments.

### 3.5. Scale-Up Gradient Elution Tests for Verification

In this work, scale-up gradient elution tests on HPD850 were conducted as in Section 2.7 to decrease the solvent consumption and to improve the desorption efficiency compared with the isocratic elution. The results of the mass, content and recovery of aucubin using gradient and isocratic elutions are listed in Table 3.

The products obtained using the isocratic mode contained an aucubin purity and recovery of 73.66% and 82.47%, respectively. In the gradient elution, the purity of aucubin was 0% in the eluate using 10% ethanol solution and was 47.05% and 63.14% with 20% and 30% ethanol solutions, respectively, for the desorption. Then, the purity of the aucubin increased remarkably when the ethanol volume fraction was increased from 40% to 80%. Because many impurities but only very small amounts of aucubin were obtained in the eluate using ethanol volume fractions in the range of 10–30%, these were used to remove impurities. In the desorption process with 40–80% ethanol volume fractions, most of the aucubin was eluted and was collected as the final product. The eluate obtained using 40–80% ethanol volume fractions was first evaporated and then dried. These contained aucubin with a purity and recovery of 79.41% and 72.92%, respectively. Compared with isocratic elution, the higher purity of aucubin in the products demonstrated the effect of gradient elution in the separation of aucubin.

### 3.6. Separation Process for Aucubin

The separation process of aucubin from the ionic liquid extraction solution of the samaras of *E. ulmoides* was investigated using a glass column that was wet-packed with the selected HPD850 resin. The 13 BV sample solution containing an initial aucubin concentration of 9.87 mg/L was passed through the glass column at 2 BV/h using a peristaltic pump at <25 °C to cause the aucubin to be fully absorbed onto the HPD850 resins. The adsorbate-laden column was first washed with 5 BV of deionized water at a flow rate of 3 BV/h to remove impurities. Then, the column was successively eluted using different ethanol volume fractions 10–80% (10–30% for removing impurities and 40–80% for collecting the aucubin) at a 2 BV/h flow rate, and 2 BV was employed for each ethanol volume fraction. The purity and recovery of aucubin in the final product were 79.41% and 72.92%, respectively.

In recent years, many processes have used ionic liquid solutions as solvents to extract target components from plant materials, in which yields are higher than those from traditional solvent ethanol solutions. While effectively isolated the target components from ionic liquid extracts was still a huge problem up to now. At present, there is a few initial attempts to adsorb the target components from the ionic liquid extracting solutions using macroporous resin as the adsorbent [28,29], and there was just a baby step along the path of getting significant isolation process, and there were still lake of the systemic study in static adsorption behaviors, adsorption isotherms, and dynamic adsorption behaviors of macroporous resin in the ionic liquid solution. In this paper, various polar types of macroporous resins were screening in terms of static adsorption and desorption taking aucubin as a target component. As a result, the HPD850 resin was selected as an adsorbent to separate aucubin from BmimBr solution, and then we executed systemic research experiments in macroporous resin static behaviors (adsorption capacity and desorption ratio, adsorption and desorption kinetic, effect of ionic liquid, adsorption isotherms, etc.) and dynamic (leakage curves, desorption curves with different desorption solvents and different flow rates) behaviors.

**Figure 6:**
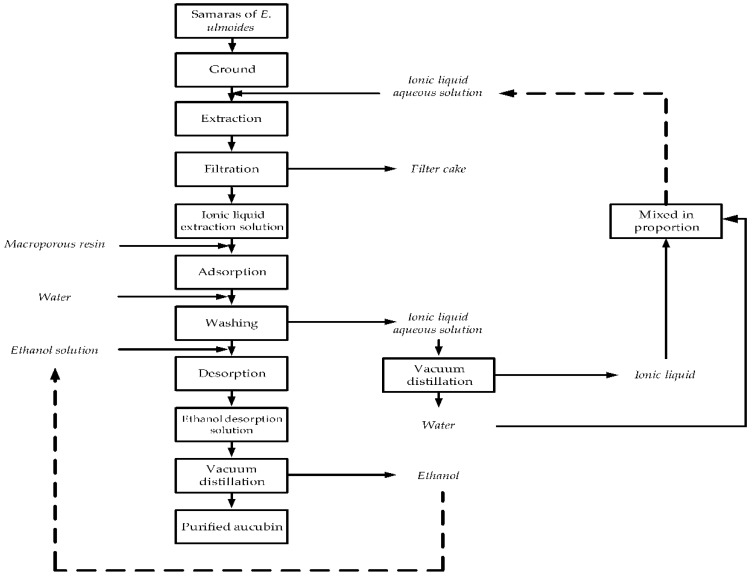
Process for the extraction, enrichment and purification of aucubin.

The use of ionic liquid solution instead of the traditional solvent ethanol solution provided higher extraction yields. Although ethanol is still used as an eluent in the separation of target components from ionic liquid solution using macroporous resins, it is used in a smaller amount than that as a solvent to extract target components from plants. In addition, the recovery of ethanol as eluent in desorption is also easier than that as extraction solvent in extraction. An scheme with the whole process was shown in Figure 6. Therefore, the use of ionic liquid solution to extract target components from plants, and then using macroporous resin as adsorbent and ethanol solution for elution in order to separate target components is a desirable approach.

## 4. Conclusions

The separation and purification of aucubin from the ionic liquid extraction solution of samaras of *E. ulmoides* with macroporous resin was successfully demonstrated in this study. Of the nine macroporous resins tested, the HPD850 resin performed the best and was selected due to its higher adsorption capacity and desorption ratio for aucubin compared to the others. In the analysis, the Langmuir isotherm model could fit and best be used to accurately describe the equilibrium adsorption experiment. In addition, the separation parameters were evaluated by dynamic adsorption and desorption experiments on columns packed with the HPD850 resin. The optimal conditions were 9.87 mg/L of initial aucubin concentration, 13 BV of sample volume, a 2 BV/h of flow rate, and a temperature of 25 °C. After absorption, 5 BV of deionized water was used to wash off impurities and the ionic liquid at a 3 BV/h flow rate. Then, the column was successively eluted using 10–80% volume fractions of ethanol at a 3 BV/h flow rate, with a 2 BV volume for each volume fraction of ethanol. The final product collected from the 40–80% ethanol eluent was found to contain an aucubin purity and recovery of 79.41% and 72.92%, respectively. The method developed in this study provides a potential means for the separation of components from ionic liquid solutions in the food and pharmaceutical industry.

## Figures and Tables

**Table 1:** Physical properties and absorption characteristics of the macroporous resins tested.

Resin	Surface Area (m^2^/g)	Average Pore Diameter (nm)	Particle Diameter (mm)	Polarity	Moisture Content (%)	Adsorption Capacity (μg/g)	Desorption Ratio (%)
HPDD	650–750	9.0–11.0	0.30–1.25	Non-polar	44.45	144.89 ± 4.24	51.06 ± 1.52
HPD100A	650–700	9.5–10.0	0.30–1.20	Non-polar	45.65	68.64 ± 1.63	66.05 ± 2.14
HPD700	650–700	8.5–9.0	0.30–1.20	Non-polar	52.31	123.56 ± 4.72	49.94 ± 1.34
DM130	500–550	9.0–10.0	0.30–1.25	Middle-polar	46.56	117.40 ± 2.37	68.30 ± 2.42
HPD750	650–700	8.5–9.0	0.30–1.20	Middle-polar	51.28	122.14 ± 4.05	67.22 ± 2.79
HPD850	1100–1300	8.5–9.5	0.30–1.20	Middle-polar	47.82	154.96 ± 4.38	79.09 ± 2.33
HPD400	500–550	7.5–8.0	0.30–1.20	Polar	48.91	125.71 ± 4.22	67.88 ± 2.28
HPD500	500–550	5.5–7.5	0.30–1.20	Polar	50.26	147.00 ± 4.53	75.08 ± 4.01
HPD600	550–600	8.0	0.30–1.20	Polar	49.55	149.70 ± 4.08	59.81 ± 1.41

**Table 2:** Langmuir and Freundlich parameters of aucubin on HPD850 resin at 5, 25 and 50 °C.

Temperature (°C)	Langmuir Equation			Freundlich Equation		
	*Q*_max_	*K*_L_	*R* ^2^	*K*_F_	1/*n*	*R* ^2^
5	0.4229	0.3187	0.9993	0.2857	0.2998	0.9199
25	0.3424	0.5991	0.9958	0.1822	0.3687	0.9749
50	0.2779	1.3265	0.9933	0.1070	0.4674	0.9143

**Table 3:** Results of elution in isocratic and gradient modes of aucubin on a column packed with HPD850 resin.

Mode	Ethanol Volume Fraction (%)	Mass of Dried Residue (mg)	Mass of Aucubin (mg)	Aucubin Purity (%)	Aucubin Recovery (%)
Isocratic mode	60	1077.42	793.63	73.66	82.47
Gradient mode	10	46.91	0.00	0.00	0.00
	20	24.44	11.5	47.05	1.20
	30	36.44	23.01	63.14	2.39
	40	112.44	92.03	81.85	9.56
	50	188.56	161.05	85.41	16.74
	60	240.56	195.56	81.29	20.32
	70	188.02	149.55	79.54	15.54
	80	81.67	46.01	56.34	4.78
	90	156.27	23.01	14.72	2.39
	Collection of 40–80	811.25	644.2	79.41	72.92
